# Relationships Between Sleepiness, Mood, and Neurocognitive Performance in Military Personnel

**DOI:** 10.3389/fneur.2019.00674

**Published:** 2019-06-27

**Authors:** F. J. Haran, Patrick Schumacher, Rachel Markwald, Justin D. Handy, Jack W. Tsao

**Affiliations:** ^1^Naval Medical Research Center, Silver Spring, MD, United States; ^2^Uniformed Services University of the Health Sciences, Bethesda, MD, United States; ^3^University of Tennessee–Knoxville, Knoxville, TN, United States; ^4^Department of Neurology, University of Tennessee Health Science Center, Memphis, TN, United States; ^5^Naval Health Research Center, San Diego, CA, United States; ^6^Stress and Motivated Behavior Institute, Syracuse, NY, United States

**Keywords:** assessment, depression, statistical methods, sleep disorders, military

## Abstract

Neurocognitive computerized assessment tools (NCATs) were developed to assist military clinicians with the tracking of recovery from injury and return to full duty decisions with a recent focus on the setting of post-concussion evaluations. However, there is limited data on the impact of deployment on neurocognitive functioning, sleepiness, and mood in healthy, non-concussed Service members. Automated Neuropsychological Assessment Metrics version 4 TBI Military (ANAM) data was obtained for a sample of active duty deployed personnel (*n* = 72) without recent history of mild traumatic brain injury (mTBI). A linear regression was conducted to examine the effects of sleepiness and mood state on neurocognitive performance. The overall multivariate regression was statistically significant. Negative mood states were the most salient predictors of neurocognitive performance with higher levels of endorsement associated with lower scores. The findings support measures of negative mood state, but not sleepiness, as relevant predictors of neurocognitive performance as measured by the ANAM. These results indicate that mood needs to be considered when reviewing neurocognitive data to ensure that appropriate clinical decisions are made; in particular for return-to-duty decisions in deployed settings after concussion recovery.

## Introduction

Traumatic brain injury (TBI), specifically mild TBI (mTBI), was considered to be the signature battlefield injury of Operations Iraqi Freedom and Enduring Freedom (1). TBI is defined by the United States Department of Defense (DoD) as “a structural or physiological disturbance of the brain, caused by an outside force that is followed by clinical symptomology” (e.g., loss of memory, slowed thinking, and confusion). It is recognized that some military Service Members (SMs) diagnosed with mTBI, especially those experiencing multiple injuries, may also experience changes in personality, sleep problems, and cognitive impairment and can increase the risk for suicide, post-traumatic stress disorder, depression, and anxiety (2, 3).

Due to these immediate and long-term impacts of mTBI it has been of importance to DoD clinical personnel to properly screen for injury and to monitor recovery from injury. Neurocognitive assessment tools (NCATs), also known as computerized neuropsychological assessment devices (CNADs) and computerized neurocognitive test (CNT) batteries, are often used to assess athletes and US military service members following an mTBI. In 2008, following the lead of the sports medicine community, Congress mandated that all SMs receive a pre-deployment neurocognitive assessment using an NCAT, the Automated Neuropsychological Assessment Metrics 4 TBI-MIL (hereafter referred to as the ANAM), prior to any deployment to a combat zone (4) for the purposes of establishing a baseline for post-injury comparative purposes. Such comparisons can be used to evaluate neurocognitive functioning to screen for deficits in cognition following an mTBI event in both the acute and post-acute phases of injury, to track recovery, and provide data to assist with return-to-duty decisions. However, it should be noted that any comparison to pre-deployment data is only valid if the assessment is an actual representation of an individual's typical neurocognitive functioning. Researchers have recently identified numerous threats to the validity of baseline assessments, including sleepiness, and mood (5).

Military personnel are especially vulnerable to any adverse effect sleepiness may have on NCAT performance as chronic insufficient sleep is prevalent among personnel deployed to combat environments (nightly average of 6.25 h) (6). Chronic insufficient sleep or sleep insufficiency occurs when sleep is insufficient to support adequate alertness, performance, and health, either because of reduced total sleep time (decreased quantity), or fragmentation of sleep by brief arousals (decreased quality). A recent meta-analysis reported sleep restriction significantly impairs cognitive functioning across a numerous cognitive domains (7). Insufficient sleep has been specifically linked to impairments in attention, reaction time, learning and memory, and decision-making (8–17). These impairments are associated with altered functioning of the dorsolateral prefrontal cortex and parietal regions of the brain (18). Research has shown that even one night of insufficient sleep can alter the connectivity of neural networks to the detriment of cognitive processes (19). Numerous studies have reported that when sleep is reduced to <7 h cognitive performance is lower in tests for vigilance, alertness, reaction time, memory, and decision-making (11, 12, 20, 21).

Abnormal mood may also challenge the validity of NCATs. The ANAM contains a self-report mood scale designed to assess several dimensions of mood (e.g., anger, anxiety, and depression) (22). Post-injury assessments may be useful for detecting changes in mood resulting from mTBI-related depression and heightened anxiety. Research has established that both depression and anxiety adversely affect cognitive processes (23, 24). Depression has been linked to decreases in both short and long-term memory, executive function, attention, and simple reaction time (23, 25). Similarly, anxiety has been linked to decreases in executive functioning and inhibition (24). Additional research focusing on SMs who served in Operations Iraqi Freedom and Enduring Freedom has indicated that combat exposure is a risk factor for both anxiety and depression (26). All the studies suggest that mood should be examined as part of any post-injury assessment.

To best of our knowledge, no study has examined the degree insufficient sleep affects performance on the ANAM4 TBI-MIL; however, research involving previous iterations of the ANAM have reported that both sleep restriction and deprivation were associated with lower scores. None of these studies reported on the relationship between insufficient sleep and the ANAM mood-scale, but Acheson et al. (27) reported that insufficient sleep resulted in increased fatigue and decreased positive mood states as indicated by the Profile of Mood States (POMS). Other studies focusing on military populations using NCATs other than the ANAM have indicated that insufficient sleep associated with high stress environments, such as high-stakes training, survival (28), and/or simulated operational environments, results in decreases in mood, and neurocognitive performance (29–32).

The aim of this study was to elucidate and compare the relationships among self-reported sleepiness, mood state, and neurocognitive performance in deployed service members as measured by the ANAM.

## Materials and Methods

### Records

The study used a retrospective cross-sectional design. A subset of healthy non-concussed SMs who received medical care for minor deployment-related orthopedic injuries at a concussion care center were extracted from an archival database containing demographic and neurocognitive assessment data from deployed Marine Corps units. Inclusion criteria was based on no history of any severity of TBI in the preceding 12 months based on the DoD and Veterans Affairs consensus criteria. History of concussion was based on the results of a self-report TBI questionnaire that is administered as part of the ANAM and determined by an endorsement of at least one of the following symptoms immediately following the injury event: feeling dazed or confused, experiencing loss of consciousness, or experiencing loss of memory for the injury. After inclusion criteria was applied, there were 72 SMs for analyses.

The original protocol was approved by the Naval Air Warfare Center Aircraft Division institutional review board, Patuxent River, MD (protocol NAWCAD.2011.0003-CR01-EMC) (33). Data from that protocol were de-identified and archived for further use. Subsequent analyses on the de-identified archival database were reviewed by the Navy Experimental Diving Unit Institutional Review Board and determined to be exempt human subject research in compliance with all applicable Federal regulations governing the protection of human subjects.

### Neurocognitive Testing

The ANAM is an automated, CNT battery that includes a sleepiness scale, mood scales, a questionnaire for self-reporting TBI, and the following subtests: Code Substitution (CDS), Matching-to-Sample (M2S), Mathematical Processing (MTH), Procedural Reaction Time (PRO), Simple Reaction Time (SRT), Code Substitution Delayed (CDD), and Simple Reaction Time Repeated (SR2) (34). Detailed descriptions of the TBI questionnaire and subtests can be found elsewhere (35). Throughput scores (mean correct responses per 60 s) from each subtest were used in all analyses. The ANAM has been shown to be a reliable and valid tool that has clinical utility as a population screening tool for the detection of neurocognitive dysfunction following a single, uncomplicated concussion within a 72-h window (36).

The sleep scale (i.e., sleepiness) is a self-reported measure of sleepiness rated on a 7-point Likert scale, with values closer to 7 indicating increased sleepiness and fatigue. The mood scale is a self-reported measure assessing seven mood dimensions, including happiness, vigor, restlessness, depression, anxiety, fatigue, and anger. Each dimension consists of six adjectives, rated on a 7-point Likert scale, with higher values representing greater degrees of endorsement of each mood state. Confirmatory factor analysis supports a 7-factor model of the mood scale, although there is evidence to suggest an alternative 2-factor model encompassing positive and negative mood states (22).

### Statistical Analyses

All analyses were performed with MATLAB 2013b (Mathworks, Natick, MA) and SPSS Version 22 (IBM, Armonk, NY).

#### Normative Data

The use of normative data is a cornerstone of neuropsychological assessment (37). An extensive normative database for the ANAM exists with over a hundred thousand data points, which stratifies the performance of healthy, non-concussed SMs according to age and gender; these data were collected as part of SMs pre-deployment baseline assessments (35).

#### Descriptive Statistics

Examinations of histograms, normality plots, and Lilliefors statistics revealed that there were minor violations of univariate normality. Thus, non-parametric statistics were used for any comparisons of means. Outliers were assessed as three times the interquartile range above the third quartile or three times below the first quartile. All outliers were removed for the analysis.

Descriptive statistics were calculated for each subtest and it was determined if any mean was outside the normal range of functioning (i.e., normal limit; defined as within the 25th to 75th percentile ranks of previously published normative ANAM data) (35). Due to the normality violations, a series of one-sample Wilcoxon signed rank tests were used to evaluate differences in sleepiness, mood scale, and subtest data between the sample and an-age matched normative sample. Effect size was evaluated using rank biserial (*r*_*sb*_) correlations and the results were interpreted using the following criteria for strength of association: small = 0.1, medium effect = 0.3, and large effect = 0.5 (38, 39). No adjustment was made for multiple comparisons. A *p*-value of ≤ 0.05 was considered significant.

#### Data Reduction

All data were converted to *Z-*scores using age-matched (21–25 years) normative data (35). The Z-score means for CDD, CDS, M2S, MTH, PRT, SRT, and SRT2 were averaged to create a neurocognitive composite score (NCS). Use of composite scores in neuropsychological testing has been reported to minimize floor and ceiling effects and reduce the risk of a Type I error (40).

Due to multicollinearity concerns within the mood scale, a principal component analysis was conducted on the seven mood subscales to reduce the dimensionality of mood data to a smaller number of latent components. As a general rule, mood dimensions that loaded below 0.4 on all extracted components were removed and the principal component analysis was repeated. The resulting uncorrelated principal components were used as predictors in regression analyses.

#### Regression Models

Multiple linear regression models with simultaneous predictor entry were run for each ANAM subtest and the ANAM composite score using sleepiness and each mood scale principal component as predictors. Collinearity diagnostics were run to ensure partial regression coefficients derived from regression analyses were estimated precisely and that the relative importance of each predictor for neurocognitive performance could be assessed reliably. Multicollinearity was measured using variable inflation factors (VIF) for each predictor. Significant multicollinearity was indicated if any VIF exceeded 4 (with values approaching 10 indicating serious multicollinearity) (41). Violations of multivariate normality were checked using histograms and QQ-plots of the standardized residuals.

## Results

### Descriptive Statistics

Table 1 presents descriptive data. The available demographic data for the overall record sample consisted of all males (100%), who were enlisted (100%), with a mean age of 25.4 (*SD* = 5.0) years. The majority of means fell within the range of normal functioning with only PRT, SRT, and SRT2 falling below the 25th percentile and anger and depression above the 75th percentile compared to the age-matched comparative norm. There was an indication toward lower scores in the deployment sample for all of the subtests, with M2S having the most prominent difference (−19%), and for vigor and happiness (one-sample Wilcoxon signed-rank tests, all *p* < 0.05). There were also indications of higher scores for sleepiness, restlessness, anxiety, anger, and fatigue.

**Table 1:** Descriptive Statistics of the ANAM Subtest, Sleepiness, and Mood Data.

**Measures**	**Normative data**		**Sample**			
	***M***	***SD***	***M***	***SD***	***p***	***ES***
**NEUROCOGNITIVE SUBTESTS**						
Code substitution delayed	48.20	15.80	42.25	17.76	0.008^*^	−0.37^††^
Code substitution	54.60	11.20	50.46	11.95	0.004^*^	−0.39^††^
Matching to sample	36.40	11.00	29.60	10.11	0.001^*^	−0.65^‡‡^
Mathematical processing	20.80	6.20	18.30	6.10	0.001^*^	−0.44^††^
Procedural reaction time	101.60	14.00	90.99^†^	21.37	0.001^*^	0.47^††^
Simple reaction time	237.80	28.40	210.69^†^	57.03	0.002^*^	0.42^††^
Simple reaction time repeated	237.40	30.60	204.27^†^	64.06	0.001^*^	−0.44^††^
Sleepiness	2.40	1.20	2.77	1.30	0.004^*^	−0.39^††^
**MOOD SCALE**						
Anger	16.20	20.00	26.16^‡^	24.41	0.010^*^	0.35^††^
Anxiety	14.80	15.00	20.46	19.92	0.049^*^	0.27^**^
Depression	12.20	17.20	17.45^‡^	19.85	0.143	0.20^**^
Fatigue	24.40	19.00	30.75	21.47	0.032^*^	0.29^**^
Happiness	64.60	21.60	54.07	25.45	0.002^*^	−0.42^††^
Restlessness	17.40	17.00	26.17	22.99	0.015^*^	0.33^††^
Vigor	57.60	19.60	48.16	20.83	0.001^*^	−0.46^††^

*M, mean; SD, standard deviation; ES, rank biserial correlations*.

* *Significant differences compared with the normative data (according to the Wilcoxon test)*.

† *Below the 25th percentile rank of normative data*.

‡ *Above the 75th percentile rank of normative data*.

** *Effect size exceeds the threshold for small effect (r_sb_ > 0.10)*.

†† *Effect size exceeds the threshold for small effect (r_sb_ > 0.30)*.

‡‡ *Effect size exceeds the threshold for small effect (r_sb_ > 0.50)*.

Table 2 presents correlations between NCS, sleepiness, and mood. There were significant associations between NCS and each of the predictor variables, with only vigor having lower than a medium effect (*r* = 0.27). There were negative correlations between neurocognitive performance and sleepiness (*r* = −0.34), restlessness (*r* = −0.40), anxiety (*r* = −0.36), depression (*r* = −0.40), anger (*r* = −0.34), and fatigue (*r* = −0.39), and positive correlations between neurocognitive performance and vigor (*r* = 0.27) and happiness (*r* = 0.38).

**Table 2:** Correlations between the ANAM sleepiness, mood, and neurocognitive composite scores.

**Measure**	**1**	**2**	**3**	**4**	**5**	**6**	**7**	**8**	**9**
1. NCS	1								
2. Sleepiness	−0.365^**^	1							
3. Vigor	0.274^*^^,^^†^	−0.615^**^^,^^‡^	1						
4. Restlessness	−0.386^**^^,^ ^†^	0.503^**^^,^ ^‡^	−0.143^,^^†^	1					
5. Depression	−0.364^**^^,^ ^†^	0.534^**^^,^ ^‡^	−0.274^*^^,^ ^†^	0.827^**^^,^ ^‡^	1				
6. Anger	−0.341^**^^,^ ^†^	0.539^**^^,^ ^‡^	−0.236^*^^,^ ^†^	0.852^**^^,^ ^‡^	0.830^**^^,^ ^‡^	1			
7. Fatigue	−0.374^**^^,^ ^†^	0.684^**^^,^ ^‡^	−0.370^**^^,^ ^†^	0.765^**^^,^ ^‡^	0.784^**^^,^ ^‡^	0.714^**^^,^ ^‡^	1		
8. Anxiety	−0.329^**^^,^ ^†^	0.337^**^^,^ ^†^	0.005	0.841^**^^,^ ^‡^	0.804^**^^,^ ^‡^	0.788^**^^,^ ^‡^	0.637^**^^,^ ^‡^	1	
9. Happiness	0.375^**^^,^ ^†^	−0.592^**^^,^ ^‡^	0.834^**^^,^ ^‡^	−0.423^**^^,^ ^†^	−0.514^**^^,^ ^‡^	−0.494^**^^,^ ^†^	−0.524^**^^,^ ^‡^	−0.274^*^^,^^†^	1

*NCS = neurocognitive composite score*.

* *Correlation is significant at the 0.05 level (2-tailed)*.

** *Correlation is significant at the 0.001 level (2-tailed)*.

† *Effect size exceeds the threshold for small effect (r > 0.10)*.

‡ *Effect size exceeds the threshold for a medium effect (r > 0.50)*.

### Data Reduction

The Kaiser-Meyer-Olkin (KMO) Test of sampling adequacy (KMO = 0.829) indicated that enough cases were present in the dataset to support the PCA. The PCA converged on a two-factor solution, with components accounting for 65 and 22% of variance, respectively. As shown in Table 3, Component 1 included negative mood states (restlessness, anxiety, depression, anger, and fatigue) whereas Component 2 included positive mood states (vigor and happiness). Vigor and happiness negatively cross-loaded onto Component 1, although this was expected given negative associations between self-reported negative and positive mood states. For multiple linear regression analyses, the negative and positive mood components, along with sleepiness, were used as predictors of neurocognitive performance in lieu of individual mood subscales.

**Table 3:** Component matrix loadings of unrotated components extracted by principal component analysis.

**Measure**	**Component Matrix**	
	**Component 1**	**Component 2**
Restlessness	**0.91**	0.26
Depression	**0.92**	0.13
Anger	**0.90**	0.15
Fatigue	**0.87**	−0.02
Anxiety	**0.83**	0.42
Vigor	−0.41	**0.88**
Happiness	−0.66	**0.70**

*Bold values indicate membership of mood scales to respective principal components*.

### Regression Models

Results of multiple linear regression analyses are presented in Table 4. Only regression models for M2S, PRT, SRT, SRT2, and NCS reached statistical significance at *p* < 0.05. Adjusted *R*^2^ values indicated that sleep and mood accounted for ~10–20% of the variability in ANAM throughput scores for M2S (Adjusted *R*^2^ = 0.11), PRT (Adjusted *R*^2^ = 0.12), SRT (Adjusted *R*^2^ = 0.19), SRT2 (Adjusted *R*^2^ = 0.22), and NCS (Adjusted *R*^2^ = 0.18). This relationship was driven by negative mood, which emerged as the only significant predictor of ANAM throughput scores in these models. Lower throughput was associated with higher negative mood state values. There were no indications of multicollinearity based on VIF and tolerance values for sleepiness (VIF = 2.10, tolerance = 0.48), negative mood (VIF = 1.82, tolerance = 0.55), and positive mood (VIF = 1.28, tolerance = 0.78), nor were there violations of multivariate normality.

**Table 4:** Summary of multiple regression models predicting ANAM test performance.

**ANAM subtest**	***F*-test**	**Adjusted *R*^**2**^**	**Sleep**	**Negative mood state**	**Positive mood state**
CDD	*F*_(3, 68)_ = 1.55, *p* = 0.208	0.02	*t* = −0.22, *p* = 0.827	*t* = −1.74, *p* = 0.087	*t* = 0.01, *p* = 0.994
CDS	*F*_(3, 68)_ = 1.47, *p* = 0.231	0.02	*t* = −0.18, *p* = 0.858	*t* = −1.35, *p* = 0.183	*t* = 0.07, *p* = 0.607
M2S	*F*_(3, 68)_ = 3.98, *p* = 0.011^*^	0.11	*t* = −0.65, *p* = 0.518	*t* = −2.07, *p* = 0.042^*^	*t* = −0.08, *p* = 0.934
MTH	*F*_(3, 68)_ = 0.29, *p* = 0.830	0.00	*t* = 0.11, *p* = 0.912	*t* = −0.12, *p* = 0.476	*t* = 0.36, *p* = 0.722
PRT	*F*_(3, 68)_ = 4.09, *p* = 0.01^**^	0.12	*t* = −0.30, *p* = 0.766	*t* = −2.24, *p* = 0.028^*^	*t* = 0.87, *p* = 0.386
SRT	*F*_(3, 68)_ = 6.45, *p* < 0.001^***^	0.19	*t* = −0.33, *p* = 0.741	*t* = −2.93, *p* = 0.005^**^	*t* = 0.81, *p* = 0.421
SRT2	*F*_(3, 68)_ = 7.59, *p* < 0.0001^***^	0.22	*t* = −0.37, *p* = 0.711	*t* = −3.22, *p* = 0.002^**^	*t* = 0.59, *p* = 0.557
NCS	*F*_(3, 68)_ = 6.16, *p* < 0.001^***^	0.18	*t* = −0.30, *p* = 0.763	*t* = −2.90, *p* = 0.005^**^	*t* = 0.66, *p* = 0.513

*CDD, Code Substitution Delayed; CDS, Code Substitution; M2S, Match to Sample; MTH, Mathematical Processing; PRT, Procedural Reaction Time; SRT, Serial Reaction Time; SRT2, Simple Reaction Time Repeated; NCS, neurocognitive composite score*.

* *Coefficient is significant at the 0.05 level (2-tailed)*.

** *Coefficient is significant at the 0.01 level (2-tailed)*.

*** *Coefficient is significant at the 0.001 level (2-tailed)*.

## Discussion

This study was the first to examine the relationships between measures of sleepiness, mood, and neurocognitive performance in deployed SMs. Elucidating the relationships between these variables is imperative for the proper interpretation of neurocognitive assessment data as negative mood and sleepiness have been reported to adversely affect neurocognitive performance (42, 43). Thus, it is especially important for clinical personnel to consider such relationships and how mood may affect performance if the data are to be used to make post-concussive injury decisions, such as return-to-duty decisions. The primary findings of the study were that measures of mood, particularly negative mood states, within the ANAM predicted neurocognitive performance during deployment. These negative effects of mood on neurocognitive performance were most pronounced for ANAM subtests indexing sensorimotor speed (i.e., SRT, SRT2, PRT), as well as an overall ANAM composite score.

This is a key finding that needs to be considered when examining post-injury data as changes in mood; specifically changes in negative mood (e.g., fatigue, depression, anxiety, irritability, and emotional lability) are commonly associated with mTBI (44–47). Most mood-related symptoms, regardless of injury severity, remain elevated for 2 weeks post-injury (48); however, depressive symptoms have been reported to remain elevated for a month or more post-injury (49). Mood-related symptomology is so common following TBI that a mood-related symptom profile is one of the suggested symptom-based profiles for combat-related mTBI (44) and one of the suggested clinical profiles for sports-related concussion (50–53). To complicate matters, mood-related symptoms are often comorbid with cognitive symptomology (e.g., problems with attention, multitasking, distractibility) (47) making it very difficult to parse out the specific effects of mood-related symptoms on neurocognitive functioning.

There were significant changes in our deployed sample compared to normative data with decreases in neurocognitive scoring and positive mood and increases in sleepiness and negative mood. The overall mood state of the sample compared to the normative data was expected due to the high levels of psychological and physical stress associated with deployment (54, 55). Recent research has linked deployment stressors to negative post-deployment psychological health outcomes including, but not limited to, increased risk of physical health problems, increased fatigue, mood swings, suicidality, irritability, anxiety disorders, major depression, and substance abuse (56). Deployment-related stressors can also be compounded by other stressors distinct to military service, including mission ambiguity, engagement ambiguity, leader climate, cultural, and situational ambiguity (56). To compound matters, the sampled Marine units are known to have high rates of repeat deployments, longer deployments, and less time between deployments, both of which are associated with decreased morale, changes in mood, decreased psychological health, stress-related work problems, and sleep dysfunction (57–59).

Studies have reported that 75% of SMs rated their sleep as worse during deployment compared to pre-deployment levels (6) and that as many as 27% of SMs returning from Operations Iraqi Freedom and Enduring Freedom have reported trouble sleeping while deployed (56). The literature is clear that service members are at risk for insufficient sleep; in terms of sleep quantity and poor sleep quality and that, these risks are aggravated by deployment, especially in redeployers (6, 59, 60). Sleep dysfunction historically has been viewed as a symptom of anxiety and/or depression with insomnia being common between the two disorders. Research has shown that there is bidirectional relationship where poorer sleep quality may be a mechanism through which work stress results in increased depression and that increased depressive symptoms may result in poorer sleep (61, 62).

Ultimately, deployment-related increases in sleepiness and negative mood states, particularly those associated with depression and anxiety, are of concern as these factors may confound the results of mTBI-related neurocognitive assessments and lead to invalid patient dispositions. It should be noted that there is currently no agreement on whether anxiety and/or depression adversely affect neurocognitive performance, with research supporting both sides (63–66). However, there are numerous ways in which mood, depression, and anxiety can be related to decreased neurocognitive performance: changes in mood (i.e., depression and anxiety) and decreased neurocognitive performance can be direct symptoms of the same injury, the mood symptoms may be a response to decreased cognitive functioning, or that mood symptoms, in and of themselves, may adversely affect cognitive functioning (67–69).

Although it is well-accepted that there is an association between sleepiness and neurocognitive performance (9, 70), sleepiness was not predictive of NCS when controlling for the presence of negative and positive mood states in regression models. This could be suggestive of mediation by the positive mood factor for the effects of sleepiness. It also could be speculated that a single Likert scale may not accurately capture changes in sleepiness that occur during deployment and a more objective measure of sleep (i.e., quality or quantity) may have more predictive utility.

### Limitations

This study has substantial limitations. First TBI history data were based on self-report rather than objective data; therefore, the results are subject to recall biases (i.e., under-reporting). Second, data were not screened for invalid test performance (i.e., poor effort) as we did not have access to all metrics produced by the ANAM. Third, there was limited demographic and service-related characteristics available in the dataset. This limited our ability to examine common confounding factors of NCS (i.e., age, education, rank, time in service, and number of deployments) in the current sample. Additionally, no clinical information regarding the presence of comorbid diagnoses, such as post-traumatic stress disorder, post-concussive syndrome, depression, anxiety, and/or adjustment disorders was available, which could also confound test results. Finally, a single Likert scale may not accurately capture changes in sleepiness that would have been hypothesized to occur during deployment because of poor sleep quality or quantity.

## Conclusion

Measures of negative mood states were found to have significant negative relationships to several neurocognitive performance domains, whereas measures of positive mood states and sleepiness did not. There were also significant differences in neurocognitive performance, sleepiness, and mood between our deployment sample and normative data. These results taken together indicate that both changes in mood, particularly negative mood states, need to be considered when reviewing data from a neurocognitive assessment, especially if the SM is deployed, to ensure that the appropriate clinical decisions are made.

## Ethics Statement

The original protocol was approved by the Naval Air Warfare Center Aircraft Division institutional review board, Patuxent River, MD (protocol NAWCAD.2011.0003-CR01-EMC) (33). Data from that protocol were de-identified and archived for further use. Subsequent analyses on the de-identified archival database were reviewed by the Navy Experimental Diving Unit Institutional Review Board and determined to be exempt human subject research in compliance with all applicable Federal regulations governing the protection of human subjects.

## Author Contributions

FH and JT contributed conception and design of the study. FH retrieved and managed the data. FH and JH performed the statistical analysis. FH, PS, and JH contributed to the initial drafting of the manuscript and wrote sections of the manuscript. All authors contributed to editing and revising of the manuscript, read and approved the submitted version.

### Conflict of Interest Statement

The authors declare that the research was conducted in the absence of any commercial or financial relationships that could be construed as a potential conflict of interest.

## Abbreviations

ANAM: Automated Neuropsychological Assessment Metrics version 4 TBI Military
DoD: Department of Defense
ImPACT: Immediate Post-Concussion Assessment and Cognitive Test
NCAT: Neurocognitive Computerized Assessment Tools
NCS: Neurocognitive Composite Score
SMs: Service Members
TBI: Traumatic Brain Injury
mTBI: Mild Traumatic Brain Injury.
